# Hypertensive disorders of pregnancy and GLP-1 receptor agonist timing: a systematic review and meta-analysis

**DOI:** 10.1007/s12020-026-04701-9

**Published:** 2026-07-31

**Authors:** Davide Pisani, Driul Lorenza, Mario Fordellone, Antonio Cerillo, Maria Fatigati, Petra Hanulíková, Alessandra Mariniello, Pasquale De Franciscis, Lucia Pasquini, Marco La Verde

**Affiliations:** 1 Department of Woman, Child and General and Specialized Surgery, Obstetrics and Gynecology Unit, University of Campania “Luigi Vanvitelli”, Naples, Italy; 2 Clinic of Obstetrics and Gynecology, “Santa Maria della Misericordia” University Hospital, Azienda Sanitaria Universitaria Friuli Centrale, Udine, 33100 Italy; 3 Department of Medicine, University of Udine, Udine, 33100 Italy; 4 Medical Statistics Unit, University of Campania Luigi Vanvitelli, Naples, Italy; 5 Institute for the Care of Mother and Child, Third Faculty of Medicine, Charles University, Prague, Czech Republic; 6 Fetal Medicine Unit, Department for Woman and Child Health, Careggi University Hospital, Florence, Italy

**Keywords:** GLP-1 receptor agonists, Preconception care, Hypertensive disorders of pregnancy, Preeclampsia, Maternal obesity, Gestational hypertension

## Abstract

**Purpose:**

Glucagon-like peptide-1 receptor agonists (GLP-1 RAs), used to treat type 2 diabetes and obesity, may improve metabolic health before conception. However, their association to hypertensive disorders of pregnancy (HDP) after periconceptional or early pregnancy exposure remains unknown.

**Methods:**

We performed a meta-analysis to investigating association between GLP-1 RAs preconception or first trimester of gestation exposure and HDP risk. Cochrane Central Register of Controlled Trials databases, ClinicalTrials.gov, PubMed, Scopus, and EMBASE databases were searched from inception through December 15, 2025. All eligible studies were observational cohorts. Case reports, reviews, editorials, and studies that lacked HDP data were excluded. We pooled odds ratios with 95% confidence intervals using a random-effects Mantel–Haenszel model. ROBINS-I tool was used to evaluate risk of bias.

**Results:**

Of 75 records identified, 3 retrospective cohort studies met inclusion criteria with 10,880 pregnancies (4942 exposed to GLP-1 RAs and 5938 unexposed). All the studies were conducted in the United States between 2014–2025 and evaluated the semaglutide, liraglutide, dulaglutide, tirzepatide, exenatide, lixisenatide, and albiglutide exposure. Two studies showed a lower HDP risks among exposed pregnant, whereas one study found a higher risk. In the pooled analysis, GLP-1 RA exposure showed a HDP risk with an OR 0.91 (OR 0.91, CI 0.57–1.47) with no statistical significance.

**Conclusion:**

Periconceptional or first-trimester exposure to GLP-1 RAs are not significantly associated with HDP risk. This available evidence is limited and indicating the need for large prospective studies to establish a possible association between GLP-1 RAs are not significantly associated with HDP risk.

## Introduction

Globally obesity among young women continue to rise [1]. Maternal obesity is associated with obstetric complications including hypertensive disorders of pregnancy (HDP), fetal growth restriction, premature birth, and congenital anomalies [2]. Gestational weight gain (GWG) management during pregnancy sometimes failed to achieve optimal results [3]. Another strategy is to optimize patients’ weight during the preconception time [4]. Glucagon-like peptide-1 receptor agonists (GLP-1 RAs) were first approved for Type 2 Diabetes Mellitus (T2DM) [5]. These drugs improve glycemic control and weight loss through different mechanism including increased insulin secretion, suppress of glucagon secretion, delayed gastric emptying, and enhanced satiety [6, 7]. Several studies explored the GLP-1 RAs impact on menstrual cyclicity, ovulation, and androgen levels in women [8]. This evidence may suggest the off-label use in women with obesity and polycystic ovary syndrome (PCOS) before pregnancy [9]. Despite these findings, studies about fetal and maternal safety are limited, and GLP-1 RAs in pregnant women are not approved [10]. GLP-1 RAs effect on embryofetal development were investigated in animal studies, and this represents the basis for preventive measures, including avoiding therapy at least 2 months prior to conception [11, 12]. However, unplanned pregnancies offered data on unintentional exposure at the beginning of pregnancy [13]. A meta-analysis found no statistically significant difference in adverse obstetric outcomes in patients exposed to GLP-1 RA before or in early pregnancy [14]. Obesity, T2DM and HDP are linked by complex physiopathogenetic pathways that act [15, 16]. Preeclampsia, HDP, and pregestational hypertension are major contributors to adverse neonatal and maternal outcomes [17]. Given the anti-inflammatory and endothelial stabilizing GLP-1 RAs role [18], one question is necessary: May GLP-1 RAs subministration, in the preconception period, impact the risk of HDP? To our knowledge, no previous meta-analysis misured the HDP risk after GLP-1 RAs use in the first trimester of pregnancy or in preconception period. This systematic review and meta-analysis examined the available evidence about pre-conceptional and first trimester exposures to the GLP-1 RAs and the risk of HDP.

## Methods

### Eligibility criteria, information sources, and search strategy

A systematic search of five databases (Cochrane Central Register of Controlled Trials databases, ClinicalTrials.gov, PubMed, Scopus, and EMBASE) was performed from inception of each database to 15 December 2025. The search terms is provided in Supplementary Table 1, and included terms: “Hypertensive Disorders of Pregnancy” and “GLP-1 Receptor Agonists”. We included studies that evaluated GLP-1 RAs exposure during the first trimester and the pre-conceptional period (until 36 months before the conception). No geographic or language restrictions were applied. Articles were excluded if they did not report HDP outcomes or if GLP-1 RAs were not used during the pre-conceptional period. Case reports, case reviews, meta-analyses, editorial letter, conference abstracts were excluded. This systematic review and metanalysis followed the PRISMA guidelines and was registered on PROSPERO, with the following code: CRD420251273858.

### Study selection

Two independent reviewers (M.F. and D.P.) screened the titles, abstracts and keywords of the identified articles. Duplicates and studies that did not meet the inclusion and exclusion criteria were excluded. Studies were excluded if they did not report the GLP-1 Ras agents or if they did not provide data on HDP. When the two reviewers did not reach a consensus, a third reviewer (M.L.V.) was consulted. Studies that met the inclusion criteria were included in the quantitative meta-analysis.

### Data extraction

Two reviewers, A.C. and M.F. independently extracted the following data from each article: (a) Study characteristics (first author, year of publication, country, study design, study population, study period, total sample size, exposed and unexposed patients and comparator group data. (b) Baseline maternal characteristics (age, pre-pregnancy body mass index (BMI), and comorbidities). (c) Exposure assessment (GLP-1 Ras type and the timing of exposure). (c) Outcomes (HDP diagnosis criteria, HDP events). A third reviewer (M.L.V.) resolved any disagreements.

### Assessment of risk of bias

Cochrane risk of bias in non-randomized studies of interventions (ROBINS-I) tool was applied to analyze the study included. The same two reviewers (A.C. and M.F.) independently assessed the risk of bias for each article using standardized forms and disagreement were resolved with by a third reviewer (M.L.V.).

### Data synthesis

We calculated the pooled estimates of the association between GLP-1RAs and HDP as odds ratios (ORs) with 95% confidence intervals (CIs). For each study, crude event counts and total sample sizes were extracted to produce 2 × 2 contingency tables. Given clinical and methodological heterogeneity (related to the different design and population included), we used a random-effects Mantel–Haenszel metanalysis. Statistical heterogeneity was assessed with the Cochran Q test and quantified with the I² statistic. Sensitivity analyses were planned to assess the robustness of the findings by sequentially excluding individual study. The analyses were conducted using RevMan version 5.4.

## Results

### Study selection and study characteristics

**Fig. 1:**
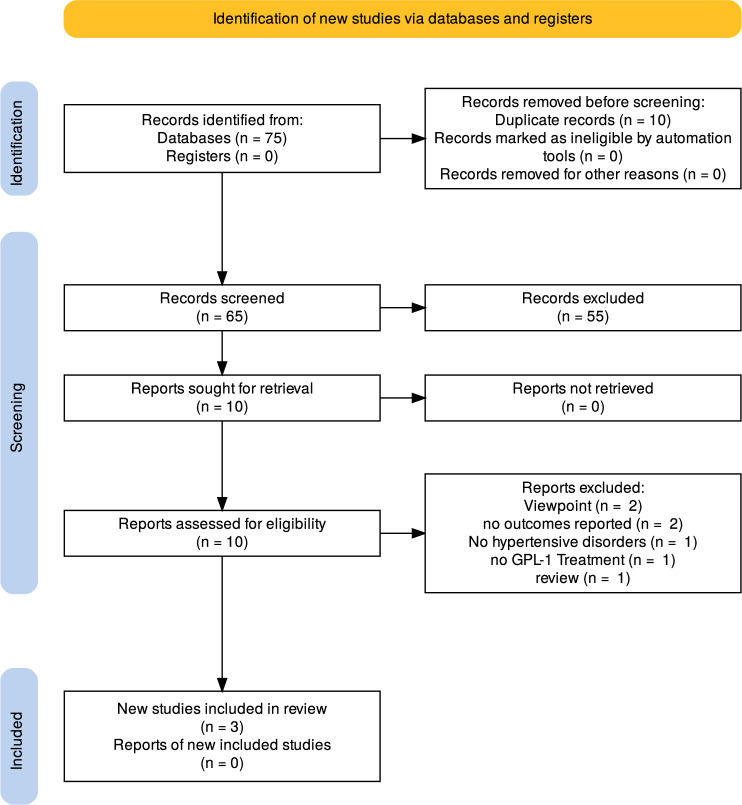
PRISMA 2020 flow diagram of the study selection process. The flowchart illustrates the identification, screening, eligibility assessment, and selection of studies in the systematic review. 75 records were identified, 65 were screened after duplicate removal, 10 full-text articles were assessed for eligibility, and 3 studies were included in the review

75 records were found (Fig. 1). After removing 10 duplicates, 65 records were screened according to title and abstract. Of the retrieved manuscripts, 61 were excluded because they were unrelated to the research question. Ten articles met the eligibility criteria and underwent full text revision. Of the ten articles, seven studies were excluded: three articles were expert opinion, letters or review; Four studies did not report the HDP outcomes or GPL-1 treatment; Three studies met the inclusion criteria and were included in quantitative-analysis [19–21] (Fig. 1). Three studies were reviewed and all were retrospective cohort studies (Table 1). The studies were conducted from 2014 to 2025 (Table 1). All three included articles were retrospective cohort studies conducted in United States of America and evaluated the pregnant exposure to GLP-1 RAs before or during early pregnancy. The total sample size of the included studies was 10,880 pregnancies: 4942 were exposed to GLP-1 RA and 5938 unexposed (Table 1). Imbroane et al. reported the largest cohort with 8534 pregnancies from January 2020 to June 2024 with exposed and unexposed pregnancies matched in a 1:1 ratio based on maternal age, race, ethnicity, and comorbid conditions [19]. Maya et al. included 1728 singleton pregnancies from June 2016 to March 2025, with 432 exposed to GLP-1-RAs and 1296 unexposed pregnancies matched by propensity-score methods in a 1:3 ratio [20]. Pondugula et al. analyzed 618 pregnancies between 2014 and 2024. In this study, 243 pregnancies were exposed while 375 pregnancies were unexposed [21]. The GLP-RAs evaluated were the semaglutide and liraglutide, dulaglutide, tirzepatide, exenatide, lixisenatide and albiglutide (Table 2). Exposure to GLP-RAs varied from up to three years before conception and to early gestation (Table 2). The definitions of gestational hypertension and hypertensive disorders of pregnancy were established using code-based International Classification of Diseases, Tenth Revision (ICD-10) diagnostic codes or clinical criteria from maternal blood pressure measurements based on available clinical data (Table 2).

**Table 1:** Study characteristics

First author, year	Country	Study design	Study population and study period	Total Sample size	Exposed	Unexposed	Comparator group
Imbroane, [19]	USA	Retrospective cohort study	Pregnant, ≥ 18 years, 1 January 2020–13 June 2024	8534	4267	4267 matched unexposed)	Pregnant witht no previous history of GLP-1 RA use. The cohort was matched 1:1, for age, race, ethnicity, and comorbidities.
Maya, [20]	USA	Retrospective cohort study	Singleton pregnancies, June 2016 - March 2025	1728*	432	1296	Unexposed pregnancies matched 1:3 via propensity score (no GLP-1 RAs from 3 years before to 90 days after conception)
Pondugula, [21]	USA	Retrospective cohort study	Patients with a delivery admission (2014–2024) and GLP-1 RA exposure up to 1 year before pregnancy	618	243	375	Two groups: 1) Pregestational diabetes (managed with non-GLP-1 RA meds); 2) Weight-management (BMI ≥ age 30)

* we considered only the HDP population without the pregestational HDP

**Table 2:** GLP-1 RA exposure assessment

First author, year	GLP-1 RA agent	Exposure time	Gestational hypertension definition
Imbroane, [19]	All formulation included in the GLP-1 RA class coding	Within 24 months before pregnancy	ICD-10 code
Maya, [20]	Semaglutide, liraglutide, dulaglutide, tirzepatide, exenatide, and lixisenatide;	From 3 years before to 90 days after conception	ACOG criteria using maternal blood pressure data; ICD-10 codes were used if clinical data were missing
Pondugula, [21]	Subcutaneous dulaglutide, exenatide, liraglutide, semaglutide, albiglutide, oral semaglutide and tirzepatide;	Up to 1 year before pregnancy or during early pregnancy	ICD-10 code

### Synthesis of the results

**Fig. 2:**
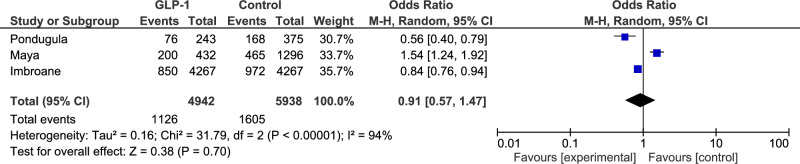
Forest plot comparing the effect of GLP-1 receptor agonist treatment versus control on the outcome of interest. The pooled random-effects meta-analysis showed no statistically significant overall effect (odds ratio [OR] = 0.91, 95% confidence interval [CI]: 0.57–1.47; *P* = 0.70), with substantial heterogeneity among the included studies (I² = 94%)

Table 3 showed the baseline maternal characteristics and hypertensive disorders of pregnancy (HDP) outcomes. Mean maternal age was similar in two studies [19, 20], with a mean values of 34.9 and 34.0 years for Imbroane et al. and Maya et al., Pondugula et al. cohort reported a greater proportion of women aged ≥ 35 years [21]. The pregnant woman’s BMI was elevated across all studies. In the Imbroane et al. cohort [19], 76.8% of participants had a BMI ≥ 25 kg/m² and the mean BMI values was 36.1 ± 6.5 kg/m² and 38.7 ± 7.8 kg/m², respectively in Maya et al. population and the diabetes subgroup of the Pondugula et al. [20, 21]. The prevalence of T2DM differed in the studies. In the Imbroane et al. cohort 37.6% of participants had T2DM, while in the Maya et al. cohort, the T2DM prevalence was 23%. In the Pondugula et al. study, 94 women in the diabetes subgroup had T2DM, while no T2DM cases were reported in the weight-management subgroup. Chronic hypertension was common in the studies included, affecting 32.5% of participants in the Imbroane et al. study and 61.2% in the diabetes subgroup of the Pondugula et al. cohort [19, 21]. Only Pondugula et al., reported the PCOS with a prevalence of 28.2% in the diabetes subgroup, and 30.0% in the weight-management subgroup. The prevalence of HDP varied among the GLP-1RA exposed pregnancies. HDP was reported in 850 pregnancies (19.9%) out of 4267 pregnancies in the Imbroane et al. study [19], in 200 of 432 pregnancies (46.3%) in the Maya et al. cohort [20] and in 43 of 103 pregnancies (41.8%) in the diabetes subgroup of the Pondugula et al. article [21]. The pooled analysis is shown in the forest plot (Fig. 2). Overall, the pooled estimate was not statistically significant. Two study indicated a lower risk of HDP (OR of 0.56 and 0.84) among women previously exposed to GLP-1 RA than among unexposed pregnancies [19, 21]. In contrast, Maya et al. showed an increased risk of HDP associated with GLP-1 RA exposure. The CI of the individual studies did not cross the line of no effect, whereas the pooled estimate (OR 0.91) crossed unity and did not reach statistical significance. Overall, the forest plot suggested a possible reduction in HDP risk in exposed pregnant, however, this finding is limited and the pooled estimate was not statistically significant.

**Table 3:** Baseline maternal characteristics and HDP outcomes

Exposure group	Maternal age, y (mean ± SD)	Prepregnancy BMI, kg/m² (mean ± SD)	T2DM, n (%)	Chronic hypertension, n (%)	PCOS, n (%)	HDP events/total (%)
Imbroane [19]	34.9 ± 6.8	76.8% ≥ 30 kg/m²*	1603 (37.6%)	1388 (32.5%)	NR	850/4267 (19.9%)
Maya [20]	34.0 ± 4.7	36.1 ± 6.5	104 (23%)	Excluded	NR	200/432 (46.3%)
Pondugula [21] (Diabetes gruop)	42.7% (≥35y)	38.7 ± 7.8	103 (100%)	63 (61.2%)	29 (28.2%)	76/243 (31.3%)
Pondugula [21] (Weight Mgmt gruop)	29.3% (≥35y)	38.3 ± 8.1	0 (0%)	48 (34.3%)	42 (30.0%)	

*NR* not reported

### Risk of bias of included studies

The Cochrane Risk of Bias in Non-randomized Studies of Interventions (ROBINS-I) tool was applied to our included studies. Given the retrospective nature of the studies included, all the articles were judged to have a moderate overall Risk of Bias. Low risk of bias was identified in four domains for Maya et al. and in three domains on seven for Pondugula et al. [19, 21], while Imbroane et al. had low risk in the domains of selection of participants and in selection of the reported result bias (Table 4).

**Table 4:** Risk of bias assessment (ROBINS-I Framework)

Bias Domain	Imbroane et al. [19]	Maya et al. [20]	Pondugula et al. [21]
1. Bias due to confounding	Moderate	Moderate	Moderate
2. Bias in selection of participants into the study	Low	Low	Low
3. Bias in classification of exposure	Moderate	Moderate	Low
4. Bias due to deviations from intended interventions	Moderate	Moderate	Moderate
5. Bias due to missing data	Moderate	Low	Low
6. Bias in measurement of outcomes	Moderate	Low	Moderate
7. Bias in selection of the reported result	Low	Low	Low
Overall Risk of Bias	Moderate	Moderate	Moderate

## Discussion

This systematic review and meta-analysis association between exposure to GLP-1RAs in pre-conceptional period or in early pregnancy and the subsequent HDP development in approximately 10,000 pregnancies. We found a substantial heterogeneity among the included studies. Two large-scale national and institutional databases suggest that such preconception GLP-1R exposure represents a protective “metabolic legacy” [19, 21], while Maya et al. proposed that cessation of these agents before conception can result in a remarkable rebound of gestational weight gain (GWG) with an increased GDM and HDP risk [20]. These findings are timely, given the emergence of a “new era” in obesity management, which GLP-1 RAs and dual agonists, such as tirzepatide, have a transformative impact on cardiovascular health in non-pregnant patients [22, 23]. The protective effects evidenced by Imbroan et al. and Pondugula et al. indicated that preconception-induced physiological optimization with GLP-1 RAs may confer long-term benefit [19, 21]. This “legacy effect” could be ascribed to the basic pathophysiology of HDP. Preeclampsia is known to be a two-step phenomenon characterized by aberrant trophoblastic invasion within the spiral arterioles followed by systemic endothelial dysfunction induced by placental hypoxia and oxidative stress [24, 25]. GLP-1 RAs decrease the systemic pro-inflammatory cytokines and enhance cyclic AMP signalling, which is integral for trophoblast migration and myometrial invasion [21, 26]. GLP-1 RAs impact on cardiovascular and renoprotective actions in T2DM is known. The positive effect of GLP-1 Ras on major adverse cardiovascular events, cardiovascular mortality, and chronic kidney disease progression has also been explored [23, 27]. Obesity increases the risk of HDP and the application of GLP-1 RAs agents to optimize metabolic health before a high-risk pregnancy may represent an important strategy. In addition to obesity, weight gain after the discontinuation of therapy may represent another HDP risk factor. Maya et al. (2025) evidenced this issue and found an 29% higher risk of HDP and a 30% increased risk of GDM in GLP-1 RAs exposed pregnancies [20]. In that article, the major HDP mechanism was an average excess weight gain in the GLP-1-RAs group compared with matched controls, and 65% of exposed patients exceeding Institute of Medicine (IOM) GWG recommendations [20]. This GWG rebound is consistent with findings from STEP-1 trial extension, in which approximately two thirds of the lost weight in non-pregnant population was regained within one year after semaglutide discontinuation [28]. Elevated GWG is an independent risk factor for HDP and GDM [29, 30]. These evidence also raise the possibility that abrupt discontinuation of GLP-1 RAs before pregnancy may create a physiological period of weight gain variability that eliminate the preconception benefits [20, 21]. Methodological divergence across the studies included may reflect different outcomes assessed and different matching strategies. Maya et al. utilized quantitative clinical data (e.g., blood pressure and laboratory data) to define HDP based on ACOG criteria [20], whereas Imbroane and Pondugula relied primarily upon ICD-10 diagnostic codes, which may suffer from underreporting and misclassification [19, 21]. Additionally, Maya et al. excluded patients with chronic hypertension. This metanalysis has different notable strengths. A major strength is that the included studies used large-scale representative data sets. This approach generated a large sample size and the propensity score matching used, supported a rigorous confounders control. The studies included different classes of GLP-1 RAs, involving all available formulations as well as other co-agonists including tirzepatide. On the other hand, several limitations should be acknowledged. All three studies were retrospective and observational and therefore cannot show causality and did not include latent confounding factors. A fundamental limitation of these large database studies is the reliance on ICD-10 diagnostic codes, which may predispose to misclassification or underreporting diagnosis. The study population was drawn only from one geographic area (USA), a high-income country, which may limit generalizability. At least, a major limitation is that the pooled estimate did not reach statistical significance. The absence of a statistically significant in association with the high heterogeneity shown the need for additional high-quality studies and did not support a causal protective effect of GLP-1 RAs on HDP.

## Conclusion

GLP-1 RAs therapy may provide a potent preconception support for the obese and T2DM women. Periconceptional GLP-1 RAs exposure was not significantly associated with HDP risk, and our findings suggested only a possible positive impact on HDP. Prospective trials are needed to quantify these risks and to build clinical strategies aimed to improve perinatal outcomes in high risk categories.

## Supplementary information

Supplementary Table Appendix1

Prisma Flowchart

Supplementary Table 1

## Data Availability

No datasets were generated or analysed during the current study.
