# Supplementary material for: Hypertensive disorders of pregnancy and GLP-1 receptor agonist timing: a systematic review and meta-analysis
Source: Endocrine. 2026 Jul 31;91(1):241. doi: 10.1007/s12020-026-04701-9 (PMC13427886; doi:10.1007/s12020-026-04701-9)
Supplement: Supplementary file 2 — Prisma Flowchart [file 12020_2026_4701_MOESM2_ESM.pdf]

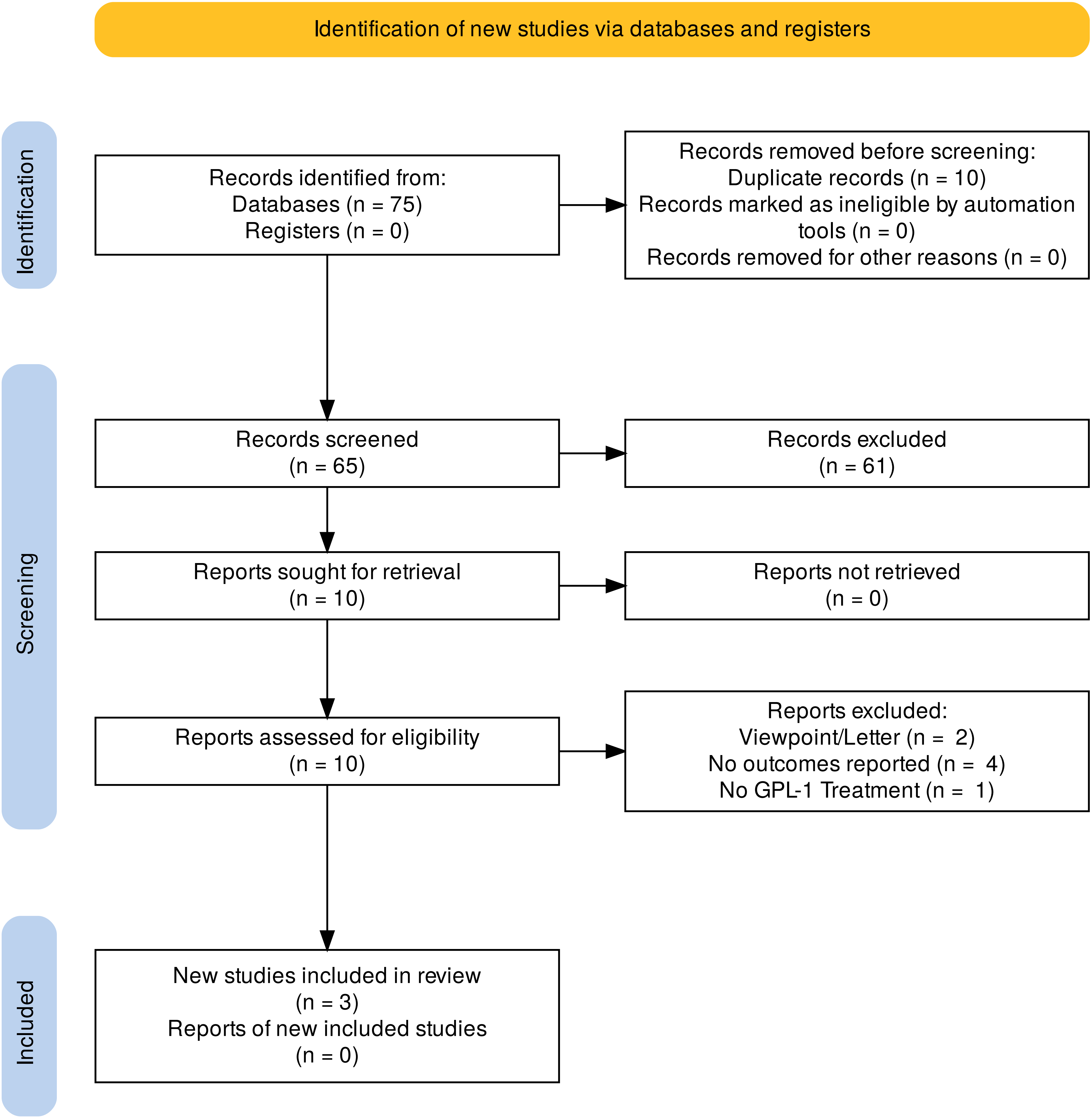
Identification of new studies via databases and registers
Identification
Screening
Included
Records identified from:
Databases (n = 75)
Registers (n = 0)
Records removed before screening:
Duplicate records (n = 10)
Records marked as ineligible by automation tools (n = 0)
Records removed for other reasons (n = 0)
Records screened
(n = 65)
Records excluded
(n = 61)
Reports sought for retrieval
(n = 10)
Reports not retrieved
(n = 0)
Reports assessed for eligibility
(n = 10)
Reports excluded:
Viewpoint/Letter (n = 2)
No outcomes reported (n = 4)
No GPL-1 Treatment (n = 1)
New studies included in review
(n = 3)
Reports of new included studies
(n = 0)
