# Supplementary material for: Hypertensive disorders of pregnancy and GLP-1 receptor agonist timing: a systematic review and meta-analysis
Source: Endocrine. 2026 Jul 31;91(1):241. doi: 10.1007/s12020-026-04701-9 (PMC13427886; doi:10.1007/s12020-026-04701-9)
Supplement: Supplementary file 3 — Supplementary Table 1 [file 12020_2026_4701_MOESM3_ESM.pdf]

Table 1. Study characteristics

First author, year	Country	Study design	Study population and study period	Total Sample size	Exposed	Unexposed	Comparator group	Association
Imbroane, 2025	USA	Retrospective cohort study	Pregnant, ≥ 18 years, 1 January 2020 – 13 June 2024	8,534	4,267	4,267 matched unexposed)	Pregnant witht no previous history of GLP-1 RA use. The cohort was matched 1:1, for age, race, ethnicity, and comorbidities.	-
Maya, 2025	USA	Retrospective cohort study	Singleton pregnancies, June 2016 - March 2025	1,728*	432	1,296	Unexposed pregnancies matched 1:3 via propensity score (no GLP-1 RAs from 3 years before to 90 days after conception)	+
Pondugula, 2025	USA	Retrospective cohort study	Patients with a delivery admission (2014–2024) and GLP-1 RA exposure up to 1 year before pregnancy	618	243	375	Two groups: 1) Pregestational diabetes (managed with non-GLP-1 RA meds); 2) Weight-management (BMI ≥age 30)	-

* we considered only the HDP population without the pregestational HDP.

* "+" indicates increased HDP risk associated with GLP-1 RA exposure; "–" indicates reduced HDP risk.
