# Supplementary material for: Hypertensive disorders of pregnancy and GLP-1 receptor agonist timing: a systematic review and meta-analysis
Source: Endocrine. 2026 Jul 31;91(1):241. doi: 10.1007/s12020-026-04701-9 (PMC13427886; doi:10.1007/s12020-026-04701-9)
Supplement: Supplementary file 1 — Supplementary Table Appendix1 [file 12020_2026_4701_MOESM1_ESM.docx]

**Table Appendix1. Detailed search strategy for systematic review.**

| **Variable** | **Search strategy** |
| --- | --- |
| Database searched | Cochrane Central Register of Controlled Trials (CENTRAL) and ClinicalTrials.gov, PubMed, Scopus and EMBASE from inception of each database until 15 December 2025. |
| Search strategy for Pubmed | ("Hypertension, Pregnancy-Induced"[Mesh] OR "Pre-Eclampsia"[Mesh] OR "Eclampsia"[Mesh] OR "HELLP Syndrome"[Mesh]) AND ("Glucagon-Like Peptide 1 Receptor Agonists"[Mesh] OR "Liraglutide"[Mesh] OR "Semaglutide"[Mesh] OR "Exenatide"[Mesh] OR "Dulaglutide"[Supplementary Concept] OR "Lixisenatide"[Supplementary Concept] OR "Tirzepatide"[Supplementary Concept]) |
| Cochrane Central Register of Controlled Trials (CENTRAL) | (("hypertensive disorder* of pregnancy" OR "pregnancy induced hypertension" OR "pregnancy-induced hypertension" OR "gestational hypertension" OR preeclampsia OR "pre-eclampsia" OR eclampsia OR "HELLP syndrome") AND ("glucagon-like peptide-1 receptor agonist*" OR "glucagon-like peptide 1 receptor agonist*" OR "GLP-1 receptor agonist*" OR liraglutide OR semaglutide OR exenatide OR dulaglutide OR lixisenatide OR tirzepatide)) |
| Scopus | ( ( "hypertensive disorder* of pregnancy" OR "pregnancy induced hypertension" OR "pregnancy-induced hypertension" OR "gestational hypertension" OR preeclampsia OR "pre-eclampsia" OR eclampsia OR "HELLP syndrome" ) AND ( "glucagon-like peptide 1 receptor agonist*" OR "GLP-1 receptor agonist*" OR liraglutide OR semaglutide OR exenatide OR dulaglutide OR lixisenatide OR tirzepatide ) ) |
| EMBASE | ('hypertensive disorder* of pregnancy' OR 'pregnancy induced hypertension'/exp OR 'gestational hypertension'/exp OR 'preeclampsia'/exp OR 'eclampsia'/exp OR 'hellp syndrome'/exp) AND ('glucagon like peptide 1 receptor agonist'/exp OR 'liraglutide'/exp OR 'semaglutide'/exp OR 'exenatide'/exp OR 'dulaglutide'/exp OR 'lixisenatide'/exp OR 'tirzepatide'/exp) |
| Clinical Trial | ((hypertensive OR "pregnancy induced hypertension" OR "pregnancy-induced hypertension" OR "gestational hypertension" OR preeclampsia OR "pre-eclampsia" OR eclampsia OR "HELLP syndrome") AND ("glucagon-like peptide-1 receptor agonist" OR "GLP-1 receptor agonist" OR liraglutide OR semaglutide OR exenatide OR dulaglutide OR lixisenatide OR tirzepatide)) |
| Other sources | The reference lists of selected articles were hand searched to identify any relevant articles. |
